# The Physician Himself and What He Should Add to His Scientific Acquirements

**Published:** 1883-04

**Authors:** 


**The Physician Himself and What He Should Add to his Scientific Acquirements.** By D. W. CATHELL, M. D. Third edition, Baltimore: Cushings & Bailey, 262 W. Baltimore Street. 1883.

The third edition of this work, following so soon after its predecessor, shows that Dr. Cathell has touched a vein of sympathy and truth among his professional brethren, from which flows a generous appreciation of his labors. The works aim to give to the young practitioner, just starting in his professional career, advice on personal questions in medical practice, in contradistinction to the strictly scientific attainments, which, it is supposed, he has acquired in the completion of the college curriculum. The book is replete with the most sensible thoughts and advice, and meets a want which every young physician must feel in the early years of his practice. We have been much interested in its perusal, and the favorable review of the second edition, which appeared in the JOURNAL, is more than justified in the more ample discussion of certain important questions in the present edition.

